# The Role of ABCB1, ABCG2, and SLC Transporters in Pharmacokinetic Parameters of Selected Drugs and Their Involvement in Drug–Drug Interactions

**DOI:** 10.3390/membranes14110223

**Published:** 2024-10-24

**Authors:** Kajetan Kiełbowski, Małgorzata Król, Estera Bakinowska, Andrzej Pawlik

**Affiliations:** Department of Physiology, Pomeranian Medical University, 70-111 Szczecin, Poland; kajetan.kielbowski@onet.pl (K.K.); malgorzatakrol246@gmail.com (M.K.); esterabakinowska@gmail.com (E.B.)

**Keywords:** drug transporters, ATP binding cassette transporters, solute carrier transporters, p-glycoprotein, drug–drug interactions

## Abstract

Membrane transporters are expressed in a wide range of tissues in the human organism. These proteins regulate the penetration of various substances such as simple ions, xenobiotics, and an extensive number of therapeutics. ABC and SLC drug transporters play a crucial role in drug absorption, distribution, and elimination. Recent decades have shown their contribution to the systemic exposure and tissue penetration of numerous drugs, thereby having an impact on pharmacokinetic and pharmacodynamic parameters. Importantly, the activity and expression of these transporters depend on numerous conditions, including intestinal microbiome profiles or health conditions. Moreover, the combined intake of other drugs or natural agents further affects the functionality of these proteins. In this review, we will discuss the involvement of ABC and SLC transporters in drug disposition. Moreover, we will present current evidence of the potential role of drug transporters as therapeutic targets.

## 1. Introduction

**Figure 1:**
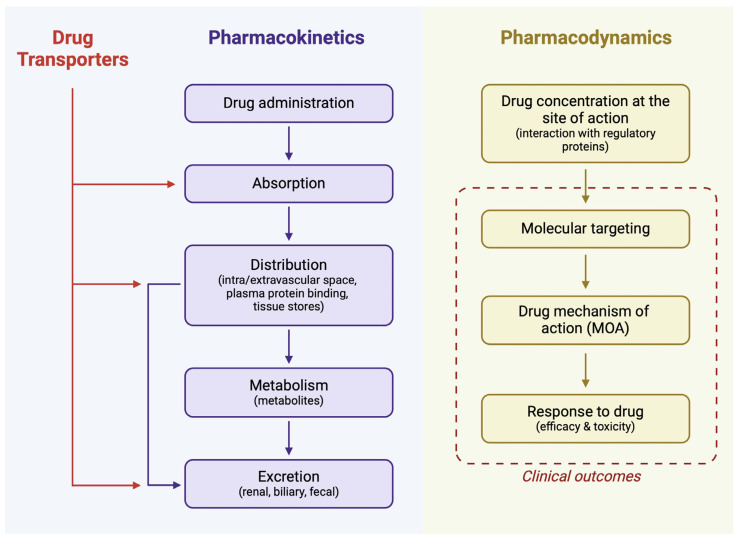
The activity of drug transporters affects pharmacokinetic parameters of drugs, which have a direct influence on their pharmacodynamic properties. Created in BioRender. Kiełbowski, K. (2024) BioRender.com/t19o310.

Pharmacology is a broad field of study relating to drugs that can be described using pharmacokinetics (PK) and pharmacodynamics (PD). PK provides information on what happens to the drug after its administration. The acronym LADME refers to the most important elements, comprising liberation, absorption, distribution, metabolism, and elimination. In contrast, PD parameters refer to the influence of the drug on an organism (Figure 1). Understanding the PK and PD parameters of drugs is crucial for them to be safely and effectively administered to humans. Age is an important factor to consider for drug administration, as PK parameters undergo developmental maturation, which has been nicely summarized by van den Anker et al. [1]. Furthermore, the processes of absorption, distribution, and elimination depend greatly upon the activity of cellular transporters. These proteins are responsible for the intake of drugs, but they are also involved in the process of efflux. Single nucleotide polymorphisms (SNPs) are a type of genetic variant that has been investigated for decades. SNPs in drug transporter genes are associated with changes in activity, which translates into modified drug distribution [2]. In this review, we will discuss the involvement of cellular membrane transporters in the distribution and bioavailability of drugs, focusing mainly on ATP-binding cassette (ABC) and solute carrier (SLC) families.

## 2. Family of Transporters—Structure and Physiological Role

### 2.1. ATP-Binding Cassette

The family of ABC proteins involves membrane transporters that are composed of nucleotide binding domains and transmembrane domains. The binding of ATP induces conformational changes that regulate the opening of the transporter. Approximately 50 ABC transporters have been identified in humans [3]. These proteins play numerous roles in human physiology; they are important transporters of ions, organic molecules, peptides, lipids, and drugs. Furthermore, they have been associated with several diseases and pathological conditions, such as cystic fibrosis (ABCC7/CFTR) or drug resistance in the case of P-glycoprotein (P-gp) [4]. As previously mentioned, these transporters perform a variety of tasks, but their involvement in drug distribution is primarily associated with drug efflux. P-gp represents a prominent member of the ABC family and the ABCB subgroup. It is involved in the distribution and elimination of therapeutic agents as it is expressed in the intestine [5], kidneys [6], and the blood–brain barrier (BBB) [7], among others sites.

**Figure 2:**
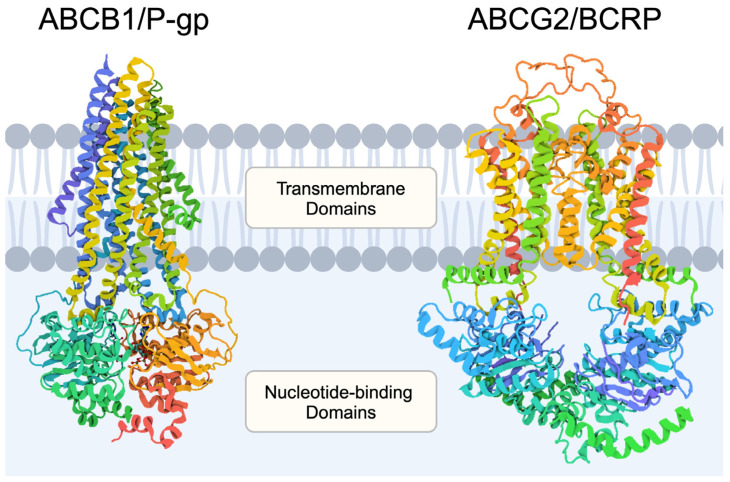
Structure of P-glycoprotein (PDB ID: 6C0V [13,14,15]) and breast cancer resistance protein (PDB ID: 6VXF [16]). Created in BioRender. Kiełbowski, K. (2024) BioRender.com/s65g631.

ABCG2, which is also known as breast cancer resistance protein (BCRP), represents another member of the ABC transporter family. It is involved in the transport of metabolites and uric acid, together with numerous drugs, including conjugates. The structure of BCRP also involves intracellular, transmembrane, and extracellular elements [8]. As the name of the transporter suggests, its presence is associated with chemoresistance in breast cancer tumors [9], including metastatic lesions [10]. The transporter is expressed in various tissues, including the placenta, gastrointestinal tract, and kidneys, among others [11]. The important physiological roles of ABCB1 and ABCG2 have been confirmed in double-knockout rats. In these animals, silencing the genes encoding the above-mentioned transporters induces significant metabolomic and transcriptomic alterations. Analyses of metabolites revealed that amino acids and lipids were significantly altered in cerebrospinal fluid and plasma, respectively. Moreover, the modification changed the transcription of hundreds of genes in the kidneys, liver, and brain. The observed effects could be a result of compensatory mechanisms associated with altered metabolome. However, other pathways were affected as well, such as pathways associated with endoplasmic reticulum stress [12]. The structure of the ABC transporters involves intracellular nucleotide-binding domains and transcellular domains (Figure 2).

### 2.2. Soluble Carrier Transporters

Solute carrier (SLC) transporters are a diverse family of membrane-bound proteins that include over 66 gene families, and over 430 secondary active transporters have been identified in humans [17]. Some scientific studies report that there are over than 600 members organized into 166 families [18] that play a crucial part in the absorption, distribution, metabolism, and elimination of various pharmacological agents [19,20]. Recent advancements in structural biology, including X-ray crystallography and nuclear magnetic resonance (NMR) spectroscopy, have significantly improved our understanding of the mechanisms and ligand interactions of SLC transporters. The two most prevalent structural folds in SLC proteins are the leucine transporter protein (LeuT)-like fold and the main facilitator superfamily (MFS) fold. The LeuT fold is made up of two five-transmembrane-helix structures, each of which has a bundle and a scaffold domain. The MFS fold is made up of two pseudo-repeats of six-transmembrane-helix structures joined by a cytoplasmic loop [21]. The structural diversity of the SLC family members is remarkable, as they comprise an array of folds that are unlikely to have evolved from one another [22]. Even in cases in which sequence similarities are modest, the folds of SLC members typically identify their processes and reveal their evolutionary linkage. The human SLC superfamily is a classic example of a distantly homologous protein family with substantial sequence diversity and relatively little structural diversity [23]. SLCs are responsible for moving a remarkably wide range of solutes through biological membranes, including medicines, amino acids, lipids, sugars, inorganic ions, and carbohydrates. The majority of these membrane proteins work as coupled symporters, or co-transporters, that propel substrate against its concentration gradient into cells by means of downhill ion (H^+^ or Na^+^) gradients. Although some members have channel-like characteristics, other members function as antiporters, which usually have a single substrate-binding site and an alternating-access mechanism of transport [24,25,26,27].

The SLC transporter superfamily comprises 66 gene families with over 430 identified secondary active transporters in humans [17]. SLC transporters, which include families such as SLC21/SLCO, SLC22, SLC15, and others, play a crucial role in the distribution and PK parameters of drugs within the human body [28].

The H^+^-coupled oligopeptide co-transporter family, also known as the solute carrier 15 (SLC15) family of peptide transporters, is a class of membrane transporters that is well known for its critical function in the cellular uptake of di- and tripeptides. These transporters, particularly PEPT1 and PEPT2, facilitate the uptake of small peptides and peptidomimetic drugs across biological membranes [29]. The extracellular domain among twelve transmembrane (TM) helices that fold into an N-terminal domain (NTD, TM1-6) and a C-terminal domain (CTD, TM7-12) play an important role in PepT1 and PepT2 functionality [30]. PepT1 is primarily responsible for mediating intestinal absorption of luminal di/tripeptides, b-lactam antibiotics (cefadroxil), and antiviral medications (valacyclovir) from overall dietary protein digestion [31,32]. SLC15A2 (PepT2) primarily permits renal tubular reuptake of di/tripeptides from ultrafiltration and brain-to-blood efflux of di/tripeptides in the choroid plexus. SLC15A3 (PHT2) and SLC15A4 (PHT1) interact with both di/tripeptides and histidine in the endolysosome membranes of immune cells, and SLC15A5 has an unknown physiological role [33]. A study by Romano at al. found that of the two SLC15 transporters expressed in thyroid follicular cells, namely PEPT2 (SLC15A2) and PHT1 (SLC15A4), only PEPT2 was involved in peptide transport at the plasma membrane, what was the first evidence that peptide transport occurs in thyroid follicular cells [34].

The SLCO/SLC21 superfamily of organic anion transporting polypeptides (OATPs) mediate the transport of a broad range of endo- and xenobiotics, leading to interindividual differences in efficacy and toxicity [35]. OATPs are classified into families and subfamilies based on amino acid sequence similarities, with 36 members identified in humans, rats, and mice [35,36]. These transporters typically have 12 transmembrane domains and function independently of sodium. Genetic variations in SLCO genes can affect OATP function and contribute to the interindividual variability of drug effects [37]. Recent studies have revealed the importance of OATPs in the pharmacology of cancer and autoimmune disorders, with certain members being overexpressed in various solid tumors and potentially influencing tumor development and disease progression [38,39].

The SLC22 transporter family is highly expressed and is involved in the regulation of homeostasis through the transportation of molecular organic compounds, including antioxidants, signaling molecules, and metabolites [40]. It can be divided into organic anion transporters (OATs), organic cation transporters (OCTs), and organic zwitterion/cation transporters (OCTNs) [41]. Recent phylogenetic analysis suggests that SLC22 transporters evolved over 450 million years ago and can be classified into at least six groups [41]. A more recent approach proposes eight functional subgroups, including four new OAT subgroups [42]. These transporters are involved in various physiological processes and diseases, such as primary systemic carnitine deficiency and renal hypouricemia [43]. Different members of the SLC22 family are expressed on the basolateral and apical surfaces of epithelial cells, where they facilitate the movement of molecules between bodily fluids and critical organs such the kidneys, liver, heart, and brain [44]. A recent study presented potential natural product (NP) interactions with other compounds at the transporter site. The researchers found that a variety of NPs, including flavonoids, vitamins, and indoles, were altered in the serum of mice with OAT1 and OAT3 knockouts. A multivariate examination of chemical characteristics indicated that OAT1- and OAT3-dependent NPs could be largely distinguished from one another. Using both protein-binding tests and in vitro transport studies, direct binding to the transporter was verified [45]. For instance, multivariate analyses confirmed the association of SLC22A3 expression with progression-free survival in pancreatic cancer patients. According to a subgroup analysis of patients receiving regimens based on nucleoside analogues, those who showed apical localization of SLC22A3 in tumor cells or negative brush border staining appeared to have a lower overall survival rate [46]. New studies have provided additional information on previously classified transporters. In the study by Redeker and Brockmöller, SLC35G3 and SLC38A10 significantly accelerated the uptake of cations such as clonidine, 3,4-methylenedioxymethamphetamine, and nicotine [47].

The structural complexity and dynamic nature of SLC transporters have long posed challenges for traditional structural biology techniques, such as X-ray crystallography [48]. The advent of cryo-electron microscopy has been a game-changer in this field. Cryo-electron microscopy (cryo-EM) has emerged as a revolutionary tool in the field of structural biology, contributing significantly to understanding of the intricate details of biomolecular structures and their functions, such as exposing multiple transporters’ binding and activation mechanisms. Moreover, this microscope has the unique ability to capture the intricate details of membrane protein structures, including their conformational changes and interactions with ligands and their complexes, without the need for crystallization. It has been applied to members of the SLC12A family, including KCCs, NKCC1, NCC, and SLC22A6, elucidating structural elements crucial for ion binding, domain interactions, and overall function [49,50]. The SLC transporter family’s druggability has been significantly impacted by the mechanisms of action that cryo-EM has helped to uncover [51].

## 3. The Involvement of Transporters in Drug Absorption, Distribution, and Elimination

### 3.1. ABC Transporters

Considering the roles of ABC proteins in drug transport and their expression in the intestine, BBB, and kidneys, the expression and functionality of these transporters affect the bioavailability and elimination of therapeutic targets. Recent studies have investigated how various conditions influence ABC transporters. P-gp, also known as ABCB1, transports a wide range of drugs, including immunosuppressants [52], natural agents [53], peptide drugs [54], and cardiological drugs [55], among others.

Experiments in rabbits have shown that the expression of P-gp is different in various segments of the intestine, thus indicating that efflux capability and drug absorbance differ between different intestinal segments [56]. P-gp molecules are transported into the apical parts of enterocytes through an interaction with Myosin Vb (Myo5B). Interestingly, in a recent study by Doolay et al. [57], the authors examined the presence of P-gp in mice and detected these transporters below the cell membrane, which potentially suggests that there is a pool of P-gp that could be rapidly inserted into the membrane [57].

**Figure 3:**
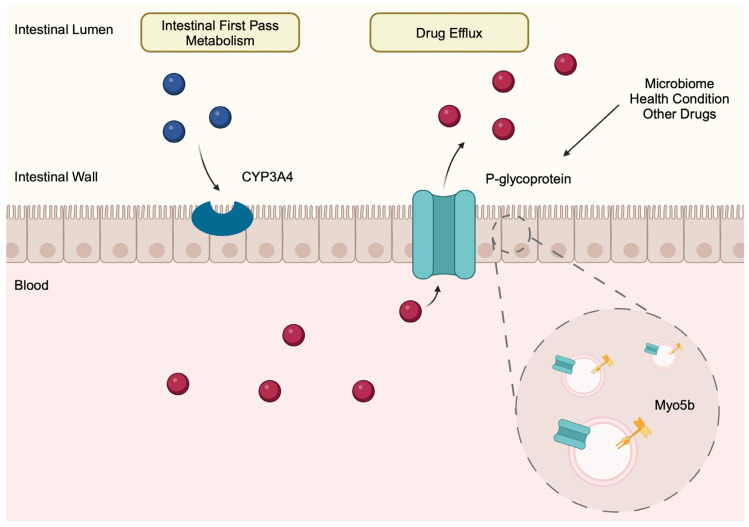
P-glycoprotein is exported to the apical parts of enterocytes, with the help of myosin Vb, where it mediates drug efflux. CYP3A4, which belongs to the family of cytochrome P450 enzymes, is also expressed in the intestinal wall, where it catalyzes first-pass metabolism and acts as a barrier for xenobiotics. Altered health condition and changed microbiome changes the activity of P-gp, which has a direct effect on drug absorption. Furthermore, the presence of other drugs and the occurrence of drug–drug interaction is associated with altered drug absorbance as well. Created in BioRender. Kiełbowski, K. (2024) BioRender.com/a30z627.

Researchers have demonstrated that various conditions affect the activity of the transporter. For instance, the expression of ABCB1 is dependent on gut microbiome composition. The extensive network of bacterial species found in the intestine play a significant role in human physiology as they affect immune responses [58]. Accordingly, altered composition of the microbiota, a condition known as gut dysbiosis, has been associated with various pathological conditions and diseases [59]. Regarding drug transporter expression, certain metabolites of the gut microbiome suppress the expression of ABCB1. However, this result was not associated with its efflux functionality [52]. A recent study proved that metabolites secreted by *Eggerthella lenta* can inhibit the drug efflux that is mediated by P-gp, thus increasing the bioavailability of digoxin [55]. A combination of several factors involving the gut microbiome could affect both transcriptional expression and functionality, thus influencing both drug efflux and absorption. For instance, some health conditions can affect the absorption efficiency of particular drugs. In an early study by Buchman and colleagues, researchers found significantly elevated expression of P-gp protein in a patient with Crohn’s disease [60]. Furthermore, in a more recent study using rats with non-alcoholic steatohepatitis, Li et al. [61] observed a higher expression of P-gp in the small intestines of these animals compared with rats from the control group. This observation was accompanied by that of decreased bioavailability of simvastatin in the study group. Importantly, these differences were not observed when the statin was administered intravenously [61]. Similarly, a higher expression of P-gp was observed in rats with portal hypertension (PH), which is associated with reduced absorbance of octreotide, a somatistatin mimic used to treat PH and PH-related complications [54]. Another condition that affects the intestinal expression of P-gp is acute kidney injury (AKI). In a rat model in which renal injury was induced by cisplatin, AKI significantly downregulated levels of P-gp located in the ileum. However, the authors made a very interesting finding: they observed that reduced expression of the P-gp transporter did not significantly affect the absorbance of Rho-123, a P-gp substrate. On the contrary, absorbance was significantly affected for the other P-gp substrate—gatifloxacin (GFLX) [62]. Thus, this important study demonstrated important physiological properties of P-gp that increase its functionality even when its intestinal expression is reduced. Interestingly, drug transporters are not the only agents that limit the entry of drugs into systemic circulation. CYP3A4 is an enzyme that is expressed in the intestine and takes part in intestinal first-pass metabolism [63]. In a study by Li and collaborators, the authors demonstrated that inhibiting CYP3A4 and not P-gp can improve the absorption of lovastatin [64] (Figure 3).

P-gp is expressed in other tissues in addition to the intestine, which is also associated with its effects on bioavailability. For instance, considering its role in drug efflux and expression in the BBB, the activity of the P-gp transporter limits the transport of drugs to the central nervous system (CNS); in the field of drug disposition, this is an undesired process as it restricts the ability of antidepressants to reach the CNS [65]. In contrast, the activity of P-gp is thought to be beneficial in Alzheimer’s disease (AD), which will be more thoroughly discussed in the following sections. Similar to the intestinal P-gp, particular conditions or substances alter the functionality of the P-gp transporter in the BBB. Copper diacetyl bis (4-methyl-3-thiosemicarbazone) (Cu(ATSM)) was recently demonstrated to enhance P-gp activity in the mouse BBB [66]. Additionally, P-gp regulates intracellular drug concentrations, as it is expressed in lymphocytes [67]. Interestingly, despite being expressed in various tissues, P-gp activity can differentially affect certain therapeutics. For instance, the phosphoinositide 3-kinase delta (PI3Kδ) inhibitor seletalisib has a very good oral bioavailability profile, which indicates low activity of the intestinal efflux mechanisms. However, P-gp significantly restricts access of the drug to the CNS, thus showing high activity towards the drug in the BBB [68]. This finding represents another obstacle for analyzing drug PK parameters, as transporters can demonstrate different activity towards therapeutics depending on the type of tissue in which they are expressed.

Regarding BCRP, the protein is responsible for the transport of numerous agents, including chemotherapeutics and tyrosine kinase inhibitors, sulfasalazine, rosuvastatin, and antileptics, among others [69]. Unlike P-gp, intestinal expression of BCRP does not seem to follow a particular trend; however, an intraindividual variability was observed [70]. Recent studies demonstrate differences between P-gp and BCRP on drug absorption. For instance, BCRP impacts the absorption of topotecan, while P-gp is thought to be linked with limited transport of the drug towards the brain [71]. By contrast, BCRP is involved in the restriction of brain distribution of zonisamide, an antileptic drug. Interestingly, the effects of drug efflux transporters can be surpassed by using a different route of administration. Specifically, intranasal delivery of antileptics is less restricted by the BCRP transporter in mice [72]. Similarly to P-gp, the functionality of BCRP seems to depend on the health condition of individuals. Chronic HCV infection affects the activity of BCRP [73], which can lead to different exposure profiles of BCRP substrates in affected patients. Another important aspect of BCRP activity is pharmacogenetics. The rs2231142 variant affects the activity of the transporter, which is associated with altered drug distribution. The acknowledgment of this clinically relevant genetic variant was described in recently published consortium guidelines linking genetic variants and statin tolerance [74]. In this document, the authors recommend a <20 mg dose of rosuvastatin as a starting dose in patients with a poor function of the ABCG2 gene (c.421AA genotype) [74].

### 3.2. SLC Transporters

SLC transporters play crucial roles in the intestinal absorption and disposition of drugs; in particular, SGLT1 (solute carrier [SLC]5A1) and SGLT2 (SLC5A2) are involved in active glucose transport in the intestine and kidneys [75,76]. Genetic disorders affecting SLC transporters can lead to conditions such as glucose–galactose malabsorption and familial renal glucosuria [77,78]. Drugs and endogenous substances are mostly taken up by hepatic SLC transporters into hepatocytes, whereas their excretion is mediated by efflux transporters [79,80]. Targeting the BBB SLC transporters holds great promise for improving medication delivery to the brain. According to Meszáros et al. [81] and Morris et al. [82], these transporters exhibit distinct patterns at the BBB and are significantly expressed in brain endothelial cells. Al-Majdoub et al. in their study show differences in transporter profiles between healthy and diseased brain tissue [83]. In vitro and in vivo models have demonstrated the enhanced absorption of cargo molecules across the BBB—and increased BBB permeability—delivered by nanoparticles targeting SLC transporters, particularly those with dual ligands [81]. The interplay between various SLC transporters in the kidney is crucial for the excretion and retention of drugs, thus impacting their overall pharmacokinetics and pharmacodynamics. The expression and function of OATs in the kidney can be altered by diseases and drug interactions, potentially leading to unexpected clinical outcomes. For instance, the dromedary camel kidney develops changes in proximal tubule brush borders after long-term dehydration. Studies suggest that ultrastructural changes in the kidney cortex and minor changes in the medulla are connected with SLCs and their mRNA expression levels [84,85]. Placental transporters, particularly those from SLC families play a very significant role in fetal drug exposure and nutrient transfer during pregnancy [86]. Understanding placental drug transport is essential for optimizing pharmacotherapy during pregnancy and predicting fetal drug exposure [87]. Recent studies show that opioid use disorder (OUD) during pregnancy can profoundly affect both the mother and fetus though changes in methylated SLCs that are associated with pathways involved in neonatal opioid withdrawal syndrome (NOWS) [88]. A vital component of the seminiferous epithelium, the blood–testis barrier (BTB) shields developing germ cells from the environment [89]. Drugs and peptides enter the seminiferous epithelium by SLC transporters like Oatp3 and Slc15a1 [89,90]. According to Touré et al. [91], the Slc26a8 (Tat1) transporter is essential for male fertility in mice and sperm terminal differentiation. A range of uptake and efflux transporters are expressed by human Sertoli cells, which regulate the flow of endogenous and foreign chemicals across the BTB [92].

Recent studies highlight the importance of SLC transporters and the connections between one another as therapeutic targets. For instance, SLC13A3 expression is elevated in human liver cancer samples. In mouse cells, β-catenin-activated liver cancer cells undergo autophagic ferroptosis and GSH depletion when SLC13A3 is silenced [93], which indicates that SLC13A3 may be a useful therapeutic target for the treatment of human liver tumors containing GOF CTNNB1 mutations. SLC38A3, SLC38A1, SLC7A6, and SLC1A5 were also linked to hepatocellular carcinoma [94].

## 4. Drug–Drug Interactions

### 4.1. ABC Transporters

When describing the effects of drug transporters on drug distribution, it is important to mention drug–drug interactions (DDIs). DDIs occur when the functionality of a transporter is modified to stimulate or suppress the absorbance of a substrate or a victim drug. In recent decades, an extensive number of studies have investigated clinical DDIs involving P-gp, which were elegantly summarized by Gessner et al. [95]. In their state-of-the-art review, these authors presented examples of DDIs associated with P-gp that occur in the intestine and kidneys [96,97,98,99,100,101,102,103,104,105] (Table 1). Researchers have recently demonstrated DDIs involving P-gp and other therapeutics. For instance, oral administration of a TRH analogue named rovatirelin together with itraconazole was associated with an increased concentration and AUC of the victim drug. Importantly, the combination of these drugs was also linked to higher concentrations of TSH and thyroid hormones [106]. Therefore, DDIs should be considered in patients receiving several drugs, which is a common situation. Studies investigating DDIs not only describe significant interactions that should bring caution to the planning of combinational therapies, but also demonstrate the safety of such treatments. In a clinical analysis, researchers examined whether remibrutinib, a Bruton’s tyrosine kinase inhibitor (BTKi), affects the bioavailability of oral contraceptives (OCs). The authors found that remibrutinib is a weak CYP3A4 and P-gp inhibitor, but its use is not associated with major DDIs, thus confirming that this BTKi can be administered with OCs [107].

These clinical data are preceded by a number of in vitro and preclinical investigations. DDIs significantly affect the bioavailability of drugs, which can enhance or weaken their desired effects. This is especially important in the case of drugs with a narrow therapeutic index or in those with a high potential for causing serious adverse events, such as anticancer therapeutics. Recently, Chu et al. found a significant P-gp-related DDI in the use of encequidar with paclitaxel [108]. Furthermore, another interaction was observed between proton pump inhibitors (PPIs) and cyclin-dependent kinase inhibitors (CDKi), which are used in patients with breast cancer. Desai and colleagues demonstrated that PPIs can block P-gp, which affects the bioavailability of anticancer therapeutics [109]. In addition to known interactions between synthetic drugs, natural agents such as flavonoids are also involved in DDIs. In rats, the combination of repaglinide with genistein was associated with significantly greater absorbance of the diabetic drug, thus demonstrating an inhibitory effect of genistein on P-gp [110].

**Figure 4:**
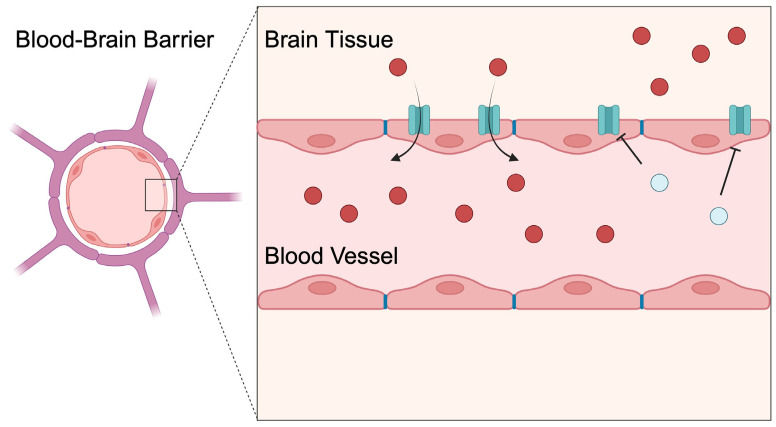
Drugs can inhibit the activity of P-glycoprotein in the blood–brain barrier to suppress the efflux mechanism and increase the concentration of desired therapeutic in the central nervous system. Hypothetically, a purposedly used DDI mechanism or P-gp inhibitor could be implemented to suppress cancer resistance. Created in BioRender. Kiełbowski, K. (2024) BioRender.com/r30a034.

An important issue to consider involves interactions that affect P-gp functionality in the BBB (Figure 4). In a recent publication by Martins et al. [111], the authors demonstrated that P-gp is involved in niraparib excretion from the CNS. Niraparib is a PARP1/2 anticancer therapeutic agent being investigated as a potential treatment agent for glioblastoma [112]. In an in vivo experiment, the use of elacridar, an ABCB1/ABCG2 inhibitor, was associated with a significantly enhanced brain-to-plasma ratio that was similar to one observed in animal models with transporter knockout [111]. Nevertheless, it should be remembered that P-gp is not only a drug transporter—it also regulates the penetration of other substances into the CNS. In an early work by Hanko et al., the authors studied rats to demonstrate that the use of P-gp inhibitors increased the penetration of bilirubin [113].

In addition, DDIs involve the activity of BCRP. BCRP-knockout mice demonstrate significantly greater plasma presence of BCRP substrates, including rosuvastatin and sulfasalazine [114], thus showing the importance of BCRP and the risk of altering substrate drug exposure after co-administration of BCRP inhibitors. Several studies examined DDIs involving statins and BCRP. Firstly, fostamatinib, a tyrosine kinase inhibitor, increases rosuvastatin concentration when they are administered together [115]. Importantly, such studies look for solutions that could be tested in clinical practice. Dong et al. [116] demonstrated that roxadustat, a drug used to treat anemia in patients with chronic kidney disease, could increase concentrations of various statins by interacting with drug transporters, including BCRP. However, the authors simulated the administration of statins 12 h after roxadustat, which inhibited the effects on statin levels when these drugs were introduced simultaneously. Another clinically relevant interaction involves rosuvastatin and finarenone, a novel mineralocorticosteroid inhibitor. By blocking three transporters—BCRP, OATP1B1, and OATP1B3—it contributes to the increased exposure of rosuvastatin. Similarly to roxadustat, separated administration of these two drugs diminished the impact of DDIs [117]. Recently, a clinical trial examined the effect of ticagrelol on rosuvastatin exposure, thus examining a popular combination used in clinical practice. Ticagrelol was found to suppress the activity of several drug transporters, including BCRP. The combination elevated the Cmax and AUC_0–∞_ of rosuvastatin 2.6-fold [118]. Another condition that could eventually lead to DDIs involving rosuvastatin is gout. The disorder is associated with a history of dyslipidemia [119]. Febuxostat, a xanthine synthase inhibitor also used in the treatment of gout, was also found to induce DDIs involving rosuvastatin and BCRP. Specifically, it increased the Cmax and AUC_0–∞_ of rosuvastatin approximately 2-fold. By contrast, there were no such findings regarding allopurinol [120]. Therefore, current evidence demonstrates an extensive interaction network between several agents and rosuvastatin, thus increasing the risk of statin-induced myopathy. With rosuvastatin being one of the most commonly used drugs for dyslipidemia, its combination with other agents may require the modification of drug administration, such as the implementation of intervals between drug intakes. These preventive measures could be introduced in patients with increased risk of developing myopathy. For instance, increased age was associated with higher rates of statin-induced myopathy [121]. Currently, researchers are analyzing potential DDIs with BCRP in a number of new drugs, which will increase knowledge about the PK parameters of BCRP substrates [122,123].

Interestingly, studies have tried to examine whether we could predict the activity of drug transporters and thus potential DDIs. Recently, riboflavin, an endogenous biomarker, was found to indicate the activity of BCRP. Hypothetically, blood levels of riboflavin could be monitored to analyze BCRP functionality and plan treatment strategies adequately to avoid the occurrence of DDIs [124]. Another approach to evaluating the activity of drug transporters is by monitoring non-coding RNA levels. MicroRNA (miRNAs) are small molecules that play a crucial role in regulating gene expression by suppressing the translation of their target mRNA. miR-138-5p and miR-146a-5p target P-gp, and their dysregulated levels were observed in patients with intractable epilepsy, a condition associated with P-gp involvement [125].

### 4.2. SLC Transporters

Over the years, studies demonstrated that various agents could inhibit the activity of SLC transporters, especially OATPs and OATs/OCTs. For instance, DDIs could probably affect patients with inflammatory disorders. OATPs/OATs/OCTs are known transporters of such drugs as non-steroid anti-inflammatory drugs (NSAIDs), as well as immunomodulatory/anticancer agents such as methotrexate (MTX) [126,127]. These groups of SLC transporters were recently found to be inhibited by ruxolitinib, a JAK inhibitor [128]. The family of JAK inhibitors is composed of several agents that are registered for the treatment of various inflammatory conditions. Thus, combinations of ruxolitinib with other immunomodulatory or anti-inflammatory drugs could potentially be associated with impacts on the exposure of the latter drugs, thus changing their profile of efficacy and toxicity. Another drug that affects MTX PK parameters is rifampicin. Hwang and colleagues [129] observed that simultaneous administration of these agents increased the following parameters: Cmax, AUC_last_, and AUC_inf_. Mechanistically, these findings involve SLC transporters, as rifampicin is an inhibitor of OATP1B1/1B3. Metformin is another important agent transported through SLC proteins. A concomitant use of metformin with isavuconazole was associated with higher plasma metformin concentrations [130]. Additionally, members of the SLC22 family mediate the transport of antiviral drugs. Inhibiting the activity of transporters while administering antivirals creates DDIs that affect the concentrations of the latter drugs. For instance, uricosuric agents, such as probenecid and lesinurad, were found to increase levels of adefovir, an anti-hepatitis B agent [131]. Similar results were observed when rats were administered with acyclovir and apigenin, a flavonoid compound [132]. Importantly, drug transporters regulate drug distribution and transportation together. Thus, a single agent can be transported through different proteins. At the same time, a single agent can be an inhibitor of several transporters. For instance, isavuconazole is considered to be an inhibitor of P-gp, OCT1, OCT2, and MATE1 [130]. As a result, administration of isavuconazole could affect the PK parameters of a variety of other drugs.

## 5. Transporters as Therapeutic Targets

### 5.1. P-Glycoprotein

In the previous sections, we have discussed modifications of P-gp functionality together with DDIs involving this transporter. A wide range of commonly used therapeutics have been found to inhibit the activity of P-gp. These findings demonstrate how P-gp activity affects drug disposition and how we can manipulate this to improve the bioavailability of certain therapeutics or introduce them through oral administration. However, the increased expression and activity of P-gp is considered an important element in cancer treatment resistance [133]. Consequently, the transporter could be targeted to reverse treatment resistance and improve therapeutic outcomes. Research in this direction has been conducted for many years. In this section, we would like to briefly describe the most recent findings on this topic. For example, a combination of chemotherapeutics with P-gp inhibitors has been studied to prevent the efflux of anticancer drugs. Recently, Zhu et al. [134] described a method to develop nanocarriers containing paclitaxel with encequidar. This method could significantly reduce tumors in vivo, and the tumor shrinkage with this treatment was greater than that observed with the use of paclitaxel alone [134]. As taxanes are administered intravenously, the ability to use oral administration would improve the quality of treatment and shorten hospital stays for treated patients. Zhou and colleagues developed a compound that is able to selectively suppress intestinal P-gp. The authors reason that intestinal suppression will increase oral bioavailability but, at the same time, sensitive tissues will be protected from paclitaxel-associated toxicities [135]. In another study, Loos and colleagues [136] showed that cabazitaxel (taxan) could be administered orally in combination with ritonavir (a CYP3A4 inhibitor), whereas the addition of elacridar increased the permeability of the brain to the chemotherapeutic agent. Moreover, studies have also begun to examine the effect of novel anticancer therapeutics on drug transporters. Recently, investigations of KRAS inhibitors were published. KRAS is an oncogene whose aberrant activity is thought to drive tumorigenesis in a wide number of malignancies. Adagrasib is a KRAS G12C inhibitor that has shown anticancer activity in malignancies harbouring the G12C mutation. This drug was recently found to suppress P-gp efflux [137]. Similar findings were recently described regarding BI-2865, a pan-KRAS inhibitor [138].

Importantly, clinical studies investigating the safety and efficacy of P-gp inhibitors are also being performed. Recently, a study examined the 72 h infusion of zosuquidar, a third-generation inhibitor of P-gp, in patients with acute myeloblastic leukaemia who were being treated with daunorubicin and cytarabine. Among 106 patients included in this trial, the overall response rate to treatment was 46%. Neurological adverse events (≥3 grade in 16% of patients) were expected based on previous trials, and these events were resolved by decreasing zosuquidar dosage or discontinuing the treatment [139].

### 5.2. BCRC Transporter

Similarly to P-gp, researchers have also focused on analyzing inhibitors of BCRP transporters. From the clinical perspective, the potential to modulate BCRP functionality is of significant importance, as it would allow clinicians to increase anticancer treatment. For instance, in an in vitro study, Sun and colleagues [140] demonstrated that suppressing BCRP could significantly improve the anticancer efficacy of lenvatinib in hepatocellular carcinoma. We have discussed studies that examined DDIs involving BCRP. A number of pharmacological agents target and suppress the activity of the transporters. In an interesting analysis by Deng et al. [141], the authors studied 232 FDA-approved agents to find BCRP inhibitors. Researchers identified 75 agents that inhibited BCRP activity, which was measured by monitoring the probe substance transport. However, developing selective ABCG2 inhibitor represents several challenges. For instance, a fungal toxin fumitremorgin C (FTC), which selectively targets BCRP, induces neurotoxic effects. By contrast, its analog, Ko143, was found to be unstable in animal plasma. However, by implementing cryo-EM techniques, Ko143 derivatives were recently found to significantly suppress the activity of BCRP [142]. Importantly, different Kol143 analogs could increase the transport through the BBB in vivo, which creates more opportunities for drugs with limited access to the brain [143]. Furthermore, tetrahydroquinoline/4,5-dihydroisoxazole hybrids were also recently proposed as potent ABCG2 inhibitors. These molecules can bind to the inhibitors’ binding region, together with a selective region for substrates [144]. Tariquidar, an agent known to inhibit both ABCB1 and ABCG2, was recently modified in a benzamide core, which formed a compound highly selective towards the BCRP transporter. Moreover, modifications involving ester moiety at the agent further improved the efficacy of these molecules and increased plasma stability [145].

### 5.3. SLC Transporters

Inhibitors of SLC transporters have been developed, ranging from constrained glutamate analogs to small molecules [146]. Numerous SLC transporters have been demonstrated to be inhibited by multi-kinase inhibitors, with OATPs, OAT3, and OCT1 being especially vulnerable [147]. Other studies have shown that plastics components such as bisphenol A (BPA) inhibit OAT3 transporter in a competitive manner [148]. There is growing interest in developing SLC transporter inhibitors for therapeutic purposes [149]. A recent study by Boeszoermenyi at al. [150] identified feeblin, which disrupts the SLC15A4-TASL adapter module. It causes interruption in the TLR7/8-IRF5 signaling pathway and as a result averts proinflammatory reactions associated with multiple autoimmune disorders. TASL is a protein associated with systemic lupus erythematosus (SLE). The lysosomal proton-coupled amino acid transporter SLC15A4 recruits TASL, which in turn activates IRF5, which consequently causes the transcription of type I interferons and cytokines [151]. Another study by Bruyère at al. revealed that the Janus kinase (JAK) inhibitor ruxolitinib inhibits the activity of OAT3 and OCT2 and blocks OAT1, OAT4, OATP1B1, OATP1B3, OATP2B1, and OCT3 [128]. Although most research has examined potential inhibitors, there is a significant interest in investigating SLC transporter activators as potential treatments for diseases associated with transporter dysfunction. Direct activators, transcriptional regulators, and trafficking modulators are some methods for modifying SLC function [152]. For example, estradiol and testosterone are activators of the expression of OATP1B1 [153], primarily found in the liver and certain tumor cells [154].

## 6. Conclusions and Future Directions

ABC and SLC transporters play a significant role in drug absorption, distribution, and elimination, thus having a crucial impact on drug bioavailability and systemic exposure. Knowledge about the physiology of drug transporters is important in studies examining PK parameters. It should be remembered that drug transporters rarely act alone, as they rather form a complex system that manages drug distribution. Furthermore, it is important to bear in mind that the impact of drug transporters on bioavailability and pharmacokinetics can be species-specific, as demonstrated by Verscheijden et al. [155]. Consequently, knowledge gained in animal models should be appropriately translated to humans. Furthermore, the occurrence of DDIs should be considered, as they can significantly affect the bioavailability of particular drugs, which translates into greater or weaker actions. Apart from altered PD, this is also linked to an altered safety profile, which needs to be properly examined. However, with increased knowledge about the safety profiles of DDIs, we could boost the bioavailability of certain agents with known poor absorbance. Thus, we could enhance the activity of several drugs or introduce an oral administration route for drugs that currently require intravenous delivery. Additionally, there is a growing field of study investigating drug transporter modulators. These agents could improve the treatment outcomes of oncological patients, benefit pharmacoeconomics, and shorten hospital stays.

## Figures and Tables

**Table 1:** Drug–drug interactions involving intestinal P-glycoprotein and their effects on pharmacokinetic parameters. Selected elements of the table reused from [95] (John Wiley and Sons; Table 1) with permission.

Victim Drug	Perpetrator Drug	PK Change of Victim Drug	Possible Change in Victim Drug Effect or Toxicity	References
Apixaban	Ketoconazole (400 mg/day for 6 days)	AUC ↑ (twofold)	Higher rate of adverse effects	[96,97]
Dabigatran etexilate	Dronedarone (1 × 400 mg)	AUC^a^ ↑ (2.1-fold)	Higher rate of adverse effects	[98]
Dabigatran etexilate	Ketoconazole (1 × 400 mg)	AUC^a^ ↑ (2.4-fold)	Higher rate of adverse effects	[98]
Edoxaban	Erythromycin (4 × 500 mg/ day for 8 days)	AUC ↑ (+85%)	Higher rate of adverse effects	[99]
Edoxaban	Cyclosporin (1 × 500 mg)	AUC ↑ (+73%)	Higher rate of adverse effects	[99]
Digoxin	Quinidine	AUC ↑ (~twofold)	Higher rate of adverse effects	[100,101,102]
Rivaroxaban	Ritonavir (2 × 600 mg/day for 5 days)	AUC ↑ (2.5-fold)	Higher rate of adverse effects	[102,103]
Apixaban	Rifampin (1 × 600 mg/day for 11 days)	AUC ↓ (−54%)	Reduced therapeutic effects	[96,102]
Dabigatran etexilate	Rifampin (1 × 600 mg/day for 7 days)	AUC^a^ ↓ (−67%)	Reduced therapeutic effects	[98,104]
Digoxin	Rifampin (1 × 600 mg/day for 10 days)	AUC ↓ (−30%)	Reduced therapeutic effects	[105]
Rovatirelin	Itraconazole (4 × 50 mg/day for 9 days)	AUC_last_ ↑ (Ratio 2.93)	Higher risk of adverse events	[106]

Note: ↑—Increased; ↓—Decreased; AUC^a^—total dabigatran AUC.

## Data Availability

No new data were created or analyzed in this study. Data sharing is not applicable to this article.

## References

[1] van den Anker J., Reed M.D., Allegaert K., Kearns G.L. (2018). Developmental Changes in Pharmacokinetics and Pharmacodynamics. J. Clin. Pharmacol..

[2] Lai Y., Varma M., Feng B., Stephens J.C., Kimoto E., El-Kattan A., Ichikawa K., Kikkawa H., Ono C., Suzuki A. (2012). Impact of drug transporter pharmacogenomics on pharmacokinetic and pharmacodynamic variability—Considerations for drug development. Expert Opin. Drug Metab. Toxicol..

[3] Locher K.P. (2016). Mechanistic diversity in ATP-binding cassette (ABC) transporters. Nat. Struct. Mol. Biol..

[4] Theodoulou F.L., Kerr I.D. (2015). ABC transporter research: Going strong 40 years on. Biochem. Soc. Trans..

[5] Fritz A., Busch D., Lapczuk J., Ostrowski M., Drozdzik M., Oswald S. (2019). Expression of clinically relevant drug-metabolizing enzymes along the human intestine and their correlation to drug transporters and nuclear receptors: An intra-subject analysis. Basic. Clin. Pharmacol. Toxicol..

[6] Kanado Y., Tsurudome Y., Omata Y., Yasukochi S., Kusunose N., Akamine T., Matsunaga N., Koyanagi S., Ohdo S. (2019). Estradiol regulation of P-glycoprotein expression in mouse kidney and human tubular epithelial cells, implication for renal clearance of drugs. Biochem. Biophys. Res. Commun..

[7] Mossel P., Arif W.M., De Souza G.S., Varela L.G., van der Weijden C.W.J., Boersma H.H., Willemsen A.T.M., Boellaard R., Elsinga P.H., Borra R.J.H. (2023). Quantification of P-glycoprotein function at the human blood-brain barrier using [(18)F]MC225 and PET. Eur. J. Nucl. Med. Mol. Imaging.

[8] Sarkadi B., Homolya L., Hegedus T. (2020). The ABCG2/BCRP transporter and its variants—From structure to pathology. FEBS Lett..

[9] Chen Y., Wang L., Zhu Y., Chen Z., Qi X., Jin L., Jin J., Hua D., Ma X. (2015). Breast cancer resistance protein (BCRP)-containing circulating microvesicles contribute to chemoresistance in breast cancer. Oncol. Lett..

[10] Uceda-Castro R., Margarido A.S., Song J.Y., de Gooijer M.C., Messal H.A., Chambers C.R., Nobis M., Citirikkaya C.H., Hahn K., Seinstra D. (2023). BCRP drives intrinsic chemoresistance in chemotherapy-naive breast cancer brain metastasis. Sci. Adv..

[11] Robey R.W., To K.K., Polgar O., Dohse M., Fetsch P., Dean M., Bates S.E. (2009). ABCG2: A perspective. Adv. Drug Deliv. Rev..

[12] Ganguly S., Finkelstein D., Shaw T.I., Michalek R.D., Zorn K.M., Ekins S., Yasuda K., Fukuda Y., Schuetz J.D., Mukherjee K. (2021). Metabolomic and transcriptomic analysis reveals endogenous substrates and metabolic adaptation in rats lacking Abcg2 and Abcb1a transporters. PLoS ONE.

[13] Kim Y., Chen J. (2018). Molecular structure of human P-glycoprotein in the ATP-bound, outward-facing conformation. Science.

[14] Berman H.M., Westbrook J., Feng Z., Gilliland G., Bhat T.N., Weissig H., Shindyalov I.N., Bourne P.E. (2000). The Protein Data Bank. Nucleic Acids Res..

[15] Sehnal D., Bittrich S., Deshpande M., Svobodova R., Berka K., Bazgier V., Velankar S., Burley S.K., Koca J., Rose A.S. (2021). Mol* Viewer: Modern web app for 3D visualization and analysis of large biomolecular structures. Nucleic Acids Res..

[16] Orlando B.J., Liao M. (2020). ABCG2 transports anticancer drugs via a closed-to-open switch. Nat. Commun..

[17] Perland E., Fredriksson R. (2017). Classification Systems of Secondary Active Transporters. Trends Pharmacol. Sci..

[18] Gyimesi G., Hediger M.A. (2022). Systematic in silico discovery of novel solute carrier-like proteins from proteomes. PLoS ONE.

[19] Kunta J.R., Sinko P.J. (2004). Intestinal drug transporters: In vivo function and clinical importance. Curr. Drug Metab..

[20] Scherrmann J.M. (2009). Transporters in absorption, distribution, and elimination. Chem. Biodivers..

[21] Bai X., Moraes T.F., Reithmeier R.A.F. (2017). Structural biology of solute carrier (SLC) membrane transport proteins. Mol. Membr. Biol..

[22] Colas C., Ung P.M., Schlessinger A. (2016). SLC Transporters: Structure, Function, and Drug Discovery. Medchemcomm.

[23] Xie T., Chi X., Huang B., Ye F., Zhou Q., Huang J. (2022). Rational exploration of fold atlas for human solute carrier proteins. Structure.

[24] Hediger M.A., Romero M.F., Peng J.B., Rolfs A., Takanaga H., Bruford E.A. (2004). The ABCs of solute carriers: Physiological, pathological and therapeutic implications of human membrane transport proteinsIntroduction. Pflugers. Arch..

[25] Fluman N., Ryan C.M., Whitelegge J.P., Bibi E. (2012). Dissection of mechanistic principles of a secondary multidrug efflux protein. Mol. Cell.

[26] Koepsell H., Endou H. (2004). The SLC22 drug transporter family. Pflugers. Arch..

[27] Schlessinger A., Geier E., Fan H., Irwin J.J., Shoichet B.K., Giacomini K.M., Sali A. (2011). Structure-based discovery of prescription drugs that interact with the norepinephrine transporter, NET. Proc. Natl. Acad. Sci. USA.

[28] Palmieri F. (2013). The mitochondrial transporter family SLC25: Identification, properties and physiopathology. Mol. Asp. Med..

[29] Ganapathy V., Miyauchi S. (2005). Transport systems for opioid peptides in mammalian tissues. AAPS J..

[30] Shen J., Hu M., Fan X., Ren Z., Portioli C., Yan X., Rong M., Zhou M. (2022). Extracellular domain of PepT1 interacts with TM1 to facilitate substrate transport. Structure.

[31] Wenzel U., Thwaites D.T., Daniel H. (1995). Stereoselective uptake of beta-lactam antibiotics by the intestinal peptide transporter. Br. J. Pharmacol..

[32] Tamai I., Nakanishi T., Hayashi K., Terao T., Sai Y., Shiraga T., Miyamoto K., Takeda E., Higashida H., Tsuji A. (1997). The predominant contribution of oligopeptide transporter PepT1 to intestinal absorption of beta-lactam antibiotics in the rat small intestine. J. Pharm. Pharmacol..

[33] Alexander S.P.H., Kelly E., Mathie A., Peters J.A., Veale E.L., Armstrong J.F., Faccenda E., Harding S.D., Pawson A.J., Sharman J.L. (2019). THE CONCISE GUIDE TO PHARMACOLOGY 2019/20: Transporters. Br. J. Pharmacol..

[34] Romano A., Barca A., Kottra G., Daniel H., Storelli C., Verri T. (2010). Functional expression of SLC15 peptide transporters in rat thyroid follicular cells. Mol. Cell. Endocrinol..

[35] Hagenbuch B., Meier P.J. (2004). Organic anion transporting polypeptides of the OATP/ SLC21 family: Phylogenetic classification as OATP/ SLCO superfamily, new nomenclature and molecular/functional properties. Pflugers. Arch..

[36] Hagenbuch B., Stieger B. (2013). The SLCO (former SLC21) superfamily of transporters. Mol. Asp. Med..

[37] Konig J. (2011). Uptake transporters of the human OATP family: Molecular characteristics, substrates, their role in drug-drug interactions, and functional consequences of polymorphisms. Handb. Exp. Pharmacol..

[38] Schulte R.R., Ho R.H. (2019). Organic Anion Transporting Polypeptides: Emerging Roles in Cancer Pharmacology. Mol. Pharmacol..

[39] Obaidat A., Roth M., Hagenbuch B. (2012). The expression and function of organic anion transporting polypeptides in normal tissues and in cancer. Annu. Rev. Pharmacol. Toxicol..

[40] Engelhart D.C., Azad P., Ali S., Granados J.C., Haddad G.G., Nigam S.K. (2020). Drosophila SLC22 Orthologs Related to OATs, OCTs, and OCTNs Regulate Development and Responsiveness to Oxidative Stress. Int. J. Mol. Sci..

[41] Zhu C., Nigam K.B., Date R.C., Bush K.T., Springer S.A., Saier M.H., Wu W., Nigam S.K. (2015). Evolutionary Analysis and Classification of OATs, OCTs, OCTNs, and Other SLC22 Transporters: Structure-Function Implications and Analysis of Sequence Motifs. PLoS ONE.

[42] Engelhart D.C., Granados J.C., Shi D., Saier M.H., Baker M.E., Abagyan R., Nigam S.K. (2020). Systems Biology Analysis Reveals Eight SLC22 Transporter Subgroups, Including OATs, OCTs, and OCTNs. Int. J. Mol. Sci..

[43] Koepsell H. (2013). The SLC22 family with transporters of organic cations, anions and zwitterions. Mol. Asp. Med..

[44] Lamhonwah A.M., Hawkins C.E., Tam C., Wong J., Mai L., Tein I. (2008). Expression patterns of the organic cation/carnitine transporter family in adult murine brain. Brain Dev..

[45] Falah K., Zhang P., Nigam A.K., Maity K., Chang G., Granados J.C., Momper J.D., Nigam S.K. (2024). In Vivo Regulation of Small Molecule Natural Products, Antioxidants, and Nutrients by OAT1 and OAT3. Nutrients.

[46] Cervenkova L., Vycital O., Bruha J., Rosendorf J., Palek R., Liska V., Daum O., Mohelnikova-Duchonova B., Soucek P. (2019). Protein expression of ABCC2 and SLC22A3 associates with prognosis of pancreatic adenocarcinoma. Sci. Rep..

[47] Redeker K.M., Brockmoller J. (2024). Several orphan solute carriers functionally identified as organic cation transporters: Substrates specificity compared with known cation transporters. J. Biol. Chem..

[48] Nair P.C., Miners J.O. (2014). Molecular dynamics simulations: From structure function relationships to drug discovery. Silico Pharmacol..

[49] Trejo F., Elizalde S., Mercado A., Gamba G., de losHeros P. (2023). SLC12A cryo-EM: Analysis of relevant ion binding sites, structural domains, and amino acids. Am. J. Physiol. Cell Physiol..

[50] Dou T., Lian T., Shu S., He Y., Jiang J. (2023). The substrate and inhibitor binding mechanism of polyspecific transporter OAT1 revealed by high-resolution cryo-EM. Nat. Struct Mol. Biol..

[51] Lees J.A., Dias J.M., Han S. (2021). Applications of Cryo-EM in small molecule and biologics drug design. Biochem. Soc. Trans..

[52] Degraeve A.L., Haufroid V., Loriot A., Gatto L., Andries V., Vereecke L., Elens L., Bindels L.B. (2023). Gut microbiome modulates tacrolimus pharmacokinetics through the transcriptional regulation of ABCB1. Microbiome.

[53] Wang M.Y., Yang M., Hou P.Y., Chen X.B., Li H.G., Yan J.X., Zhang J., Zhang Y.W., Wu X.H. (2018). Intestinal absorption of pallidifloside D are limited by P-glycoprotein in mice. Xenobiotica.

[54] Sun X., Tang S., Hou B., Duan Z., Liu Z., Li Y., He S., Wang Q., Chang Q. (2021). Overexpression of P-glycoprotein, MRP2, and CYP3A4 impairs intestinal absorption of octreotide in rats with portal hypertension. BMC Gastroenterol..

[55] Kyaw T.S., Zhang C., Sandy M., Trepka K., Zhang S., Ramirez Hernandez L.A., Ramirez L., Goh J.J.N., Yu K., Dimassa V. (2024). Human gut Actinobacteria boost drug absorption by secreting P-glycoprotein ATPase inhibitors. iScience.

[56] Ashmawy S.M., El-Gizawy S.A., El Maghraby G.M., Osman M.A. (2019). Regional difference in intestinal drug absorption as a measure for the potential effect of P-glycoprotein efflux transporters. J. Pharm. Pharmacol..

[57] Dooley S.A., Kolobova E., Burman A., Kaji I., Digrazia J.R., Stubler R., Goldstein A., Packirisamy C., Coutts A.W., Saqui-Salces M. (2024). Myosin Vb Traffics P-glycoprotein to the Apical Membrane of Intestinal Epithelial Cells. Gastroenterology.

[58] Takiishi T., Fenero C.I.M., Camara N.O.S. (2017). Intestinal barrier and gut microbiota: Shaping our immune responses throughout life. Tissue Barriers.

[59] Zhang T., Gao G., Kwok L.Y., Sun Z. (2023). Gut microbiome-targeted therapies for Alzheimer’s disease. Gut Microbes.

[60] Buchman A.L., Paine M.F., Wallin A., Ludington S.S. (2005). A higher dose requirement of tacrolimus in active Crohn’s disease may be related to a high intestinal P-glycoprotein content. Dig. Dis. Sci..

[61] Li Z., Zhang J., Zhang Y., Zhou L., Zhao J., Lyu Y., Poon L.H., Lin Z., To K.K.W., Yan X. (2021). Intestinal absorption and hepatic elimination of drugs in high-fat high-cholesterol diet-induced non-alcoholic steatohepatitis rats: Exemplified by simvastatin. Br. J. Pharmacol..

[62] Takeda F., Oda M., Terasaki M., Ichimura Y., Kojima H., Saitoh H. (2021). Downregulated expression of intestinal P-glycoprotein in rats with cisplatin-induced acute kidney injury causes amplification of its transport capacity to maintain “gatekeeper” function. Toxicol. Appl. Pharmacol..

[63] Thummel K.E. (2007). Gut instincts: CYP3A4 and intestinal drug metabolism. J. Clin. Investig..

[64] Li J., Di L., Cheng X., Ji W., Piao H., Cheng G., Zou M. (2020). The characteristics and mechanism of co-administration of lovastatin solid dispersion with kaempferol to increase oral bioavailability. Xenobiotica.

[65] Spieler D., Namendorf C., Namendorf T., Uhr M. (2019). abcb1ab p-glycoprotein is involved in the uptake of the novel antidepressant vortioxetine into the brain of mice. J. Psychiatr. Res..

[66] Pyun J., Koay H., Runwal P., Mawal C., Bush A.I., Pan Y., Donnelly P.S., Short J.L., Nicolazzo J.A. (2023). Cu(ATSM) Increases P-Glycoprotein Expression and Function at the Blood-Brain Barrier in C57BL6/J Mice. Pharmaceutics.

[67] Ito K., Naoi M., Nishiyama K., Kudo T., Tsuda Y., MacLean C., Ishiguro N. (2023). Impact of P-glycoprotein on intracellular drug concentration in peripheral blood mononuclear cells and K562 cells. Drug. Metab. Pharmacokinet.

[68] Nicolas J.M., Chanteux H., Nicolai J., Brouta F., Viot D., Rosseels M.L., Gillent E., Bonnaillie P., Mathy F.X., Long J. (2020). Role of P-glycoprotein in the brain disposition of seletalisib: Evaluation of the potential for drug-drug interactions. Eur. J. Pharm. Sci..

[69] Mao Q., Unadkat J.D. (2015). Role of the breast cancer resistance protein (BCRP/ABCG2) in drug transport--an update. AAPS J..

[70] Bruyere A., Decleves X., Bouzom F., Ball K., Marques C., Treton X., Pocard M., Valleur P., Bouhnik Y., Panis Y. (2010). Effect of variations in the amounts of P-glycoprotein (ABCB1), BCRP (ABCG2) and CYP3A4 along the human small intestine on PBPK models for predicting intestinal first pass. Mol. Pharm..

[71] Kawahara I., Nishikawa S., Yamamoto A., Kono Y., Fujita T. (2020). Assessment of contribution of BCRP to intestinal absorption of various drugs using portal-systemic blood concentration difference model in mice. Pharmacol. Res. Perspect..

[72] Goncalves J., Silva S., Gouveia F., Bicker J., Falcao A., Alves G., Fortuna A. (2021). A combo-strategy to improve brain delivery of antiepileptic drugs: Focus on BCRP and intranasal administration. Int. J. Pharm..

[73] Pippa L.F., Vieira C.P., Caris J.A., Rocha A., Marques M.P., Garcia C.P., Rezende R.E.F., Lanchote V.L. (2023). Effect of Chronic Hepatitis C on the Activity of the Membrane Transporters P-gp and OATP1B1/BCRP on Patients With Different Stages of Hepatic Fibrosis. Clin. Pharmacol. Ther..

[74] Cooper-DeHoff R.M., Niemi M., Ramsey L.B., Luzum J.A., Tarkiainen E.K., Straka R.J., Gong L., Tuteja S., Wilke R.A., Wadelius M. (2022). The Clinical Pharmacogenetics Implementation Consortium Guideline for SLCO1B1, ABCG2, and CYP2C9 genotypes and Statin-Associated Musculoskeletal Symptoms. Clin. Pharmacol. Ther..

[75] Poulsen S.B., Fenton R.A., Rieg T. (2015). Sodium-glucose cotransport. Curr. Opin. Nephrol. Hypertens..

[76] Wright E.M., Hirayama B.A., Loo D.F. (2007). Active sugar transport in health and disease. J. Intern. Med..

[77] Elsas L.J., Rosenberg L.E. (1969). Familial renal glycosuria: A genetic reappraisal of hexose transport by kidney and intestine. J. Clin. Investig..

[78] Meeuwisse G.W., Melin K. (1969). Glucose-galactose malabsorption. A clinical study of 6 cases. Acta Pædiatrica.

[79] Kalliokoski A., Niemi M. (2009). Impact of OATP transporters on pharmacokinetics. Br. J. Pharmacol..

[80] Otsuka M., Matsumoto T., Morimoto R., Arioka S., Omote H., Moriyama Y. (2005). A human transporter protein that mediates the final excretion step for toxic organic cations. Proc. Natl. Acad. Sci. USA.

[81] Meszaros M., Porkolab G., Kiss L., Pilbat A.M., Kota Z., Kupihar Z., Keri A., Galbacs G., Siklos L., Toth A. (2018). Niosomes decorated with dual ligands targeting brain endothelial transporters increase cargo penetration across the blood-brain barrier. Eur. J. Pharm. Sci..

[82] Morris M.E., Rodriguez-Cruz V., Felmlee M.A. (2017). SLC and ABC Transporters: Expression, Localization, and Species Differences at the Blood-Brain and the Blood-Cerebrospinal Fluid Barriers. AAPS J..

[83] Al-Majdoub Z.M., Al Feteisi H., Achour B., Warwood S., Neuhoff S., Rostami-Hodjegan A., Barber J. (2019). Proteomic Quantification of Human Blood-Brain Barrier SLC and ABC Transporters in Healthy Individuals and Dementia Patients. Mol. Pharm..

[84] Damir H.A., Ali M.A., Adem M.A., Amir N., Ali O.M., Tariq S., Adeghate E., Greenwood M.P., Lin P., Alvira-Iraizoz F. (2024). Effects of long-term dehydration and quick rehydration on the camel kidney: Pathological changes and modulation of the expression of solute carrier proteins and aquaporins. BMC Vet. Res..

[85] Huo X., Liu K. (2018). Renal organic anion transporters in drug-drug interactions and diseases. Eur. J. Pharm. Sci..

[86] Walker N., Filis P., Soffientini U., Bellingham M., O’Shaughnessy P.J., Fowler P.A. (2017). Placental transporter localization and expression in the Human: The importance of species, sex, and gestational age differencesdagger. Biol. Reprod..

[87] Mao Q., Chen X. (2022). An update on placental drug transport and its relevance to fetal drug exposure. Med. Rev..

[88] Radhakrishna U., Radhakrishnan R., Uppala L.V., Muvvala S.B., Prajapati J., Rawal R.M., Bahado-Singh R.O., Sadhasivam S. (2024). Prenatal opioid exposure significantly impacts placental protein kinase C (PKC) and drug transporters, leading to drug resistance and neonatal opioid withdrawal syndrome. Front. Neurosci..

[89] Su L., Mruk D.D., Lee W.M., Cheng C.Y. (2011). Drug transporters and blood--testis barrier function. J. Endocrinol..

[90] Su L., Zhang Y., Cheng Y.C., Lee W.M., Ye K., Hu D. (2015). Slc15a1 is involved in the transport of synthetic F5-peptide into the seminiferous epithelium in adult rat testes. Sci. Rep..

[91] Toure A., Lhuillier P., Gossen J.A., Kuil C.W., Lhote D., Jegou B., Escalier D., Gacon G. (2007). The testis anion transporter 1 (Slc26a8) is required for sperm terminal differentiation and male fertility in the mouse. Hum. Mol. Genet..

[92] Hau R.K., Wright S.H., Cherrington N.J. (2023). Drug Transporters at the Human Blood-Testis Barrier. Drug. Metab. Dispos..

[93] Zhao W., Wang X., Han L., Zhang C., Wang C., Kong D., Zhang M., Xu T., Li G., Hu G. (2024). SLC13A3 is a major effector downstream of activated beta-catenin in liver cancer pathogenesis. Nat. Commun..

[94] Tambay V., Raymond V.A., Voisin L., Meloche S., Bilodeau M. (2024). Reprogramming of Glutamine Amino Acid Transporters Expression and Prognostic Significance in Hepatocellular Carcinoma. Int. J. Mol. Sci..

[95] Gessner A., Konig J., Fromm M.F. (2019). Clinical Aspects of Transporter-Mediated Drug-Drug Interactions. Clin. Pharmacol. Ther..

[96] Squibb B.-M. SmPC Eliquis 5 mg Filmtabletten. https://www.medicines.org.uk/emc/product/2878.

[97] Frost C.E., Byon W., Song Y., Wang J., Schuster A.E., Boyd R.A., Zhang D., Yu Z., Dias C., Shenker A. (2015). Effect of ketoconazole and diltiazem on the pharmacokinetics of apixaban, an oral direct factor Xa inhibitor. Br. J. Clin. Pharmacol..

[98] Ingelheim B. SmPC Pradaxa 110 mg Hartkapseln. https://www.medicines.org.uk/emc/product/6229.

[99] Sankyo D. SmPC Lixiana 60 mg Filmtabletten. https://www.medicines.org.uk/emc/product/6905.

[100] Igel S., Drescher S., Murdter T., Hofmann U., Heinkele G., Tegude H., Glaeser H., Brenner S.S., Somogyi A.A., Omari T. (2007). Increased absorption of digoxin from the human jejunum due to inhibition of intestinal transporter-mediated efflux. Clin. Pharmacokinet..

[101] Leahey E.B., Reiffel J.A., Drusin R.E., Heissenbuttel R.H., Lovejoy W.P., Bigger J.T. (1978). Interaction between quinidine and digoxin. JAMA.

[102] Preston C.L. (2016). Stockley’s Drug Interactions.

[103] Bayer SmPC Xarelto 10 mg Filmtabletten. https://www.medicines.org.uk/emc/product/6402.

[104] Hartter S., Koenen-Bergmann M., Sharma A., Nehmiz G., Lemke U., Timmer W., Reilly P.A. (2012). Decrease in the oral bioavailability of dabigatran etexilate after co-medication with rifampicin. Br. J. Clin. Pharmacol..

[105] Greiner B., Eichelbaum M., Fritz P., Kreichgauer H.P., von Richter O., Zundler J., Kroemer H.K. (1999). The role of intestinal P-glycoprotein in the interaction of digoxin and rifampin. J. Clin. Investig..

[106] Kobayashi K., Abe Y., Kawai A., Furihata T., Endo T., Takeda H. (2020). Pharmacokinetic Drug Interactions of an Orally Available TRH Analog (Rovatirelin) With a CYP3A4/5 and P-Glycoprotein Inhibitor (Itraconazole). J. Clin. Pharmacol..

[107] Schiller H., Huth F., Schuhler C., Drollmann A., Kaul M., Woessner R., Shah B., Weis W., End P. (2022). Novel Bruton’s tyrosine kinase inhibitor remibrutinib: Assessment of drug-drug interaction potential as a perpetrator of cytochrome P450 enzymes and drug transporters and the impact of covalent binding on possible drug interactions. Eur. J. Pharm. Sci..

[108] Chu J., Panfen E., Wang L., Marino A., Chen X.Q., Fancher R.M., Landage R., Patil O., Desai S.D., Shah D. (2023). Evaluation of Encequidar as An Intestinal P-gp and BCRP Specific Inhibitor to Assess the Role of Intestinal P-gp and BCRP in Drug-Drug Interactions. Pharm. Res..

[109] Desai M.P., Harish Patil P., Vullendula S.K.A., Birangal S., Shenoy G.G., Rao M., Dengale S.J., Bhat K., Channabasavaiah J.P. (2023). Molecular Insights into the Mechanism of Modulatory Effects of Proton Pump Inhibitors on P-glycoprotein Mediated Drug Transport of Palbociclib and Ribociclib. Curr. Drug Metab..

[110] Jin H., Zhu Y., Wang C., Meng Q., Wu J., Sun P., Ma X., Sun H., Huo X., Liu K. (2020). Molecular pharmacokinetic mechanism of the drug-drug interaction between genistein and repaglinide mediated by P-gp. Biomed. Pharmacother..

[111] ML F.M., Loos N.H.C., Mucuk S., de Jong D., Lebre M.C., Rosing H., Tibben M., Beijnen J.H., Schinkel A.H. (2021). P-Glycoprotein (ABCB1/MDR1) Controls Brain Penetration and Intestinal Disposition of the PARP1/2 Inhibitor Niraparib. Mol. Pharm..

[112] Jones A.B., Tuy K., Hawkins C.C., Quinn C.H., Saad J., Gary S.E., Beierle E.A., Ding L., Rochlin K.M., Lamb L.S. (2024). Temozolomide and the PARP Inhibitor Niraparib Enhance Expression of Natural Killer Group 2D Ligand ULBP1 and Gamma-Delta T Cell Cytotoxicity in Glioblastoma. Cancers.

[113] Hanko E., Tommarello S., Watchko J.F., Hansen T.W. (2003). Administration of drugs known to inhibit P-glycoprotein increases brain bilirubin and alters the regional distribution of bilirubin in rat brain. Pediatr. Res..

[114] Karibe T., Hagihara-Nakagomi R., Abe K., Imaoka T., Mikkaichi T., Yasuda S., Hirouchi M., Watanabe N., Okudaira N., Izumi T. (2015). Evaluation of the usefulness of breast cancer resistance protein (BCRP) knockout mice and BCRP inhibitor-treated monkeys to estimate the clinical impact of BCRP modulation on the pharmacokinetics of BCRP substrates. Pharm. Res..

[115] Elsby R., Martin P., Surry D., Sharma P., Fenner K. (2016). Solitary Inhibition of the Breast Cancer Resistance Protein Efflux Transporter Results in a Clinically Significant Drug-Drug Interaction with Rosuvastatin by Causing up to a 2-Fold Increase in Statin Exposure. Drug Metab. Dispos..

[116] Dong J., Prieto Garcia L., Huang Y., Tang W., Lundahl A., Elebring M., Ahlstrom C., Vildhede A., Sjogren E., Nagard M. (2023). Understanding Statin-Roxadustat Drug-Drug-Disease Interaction Using Physiologically-Based Pharmacokinetic Modeling. Clin. Pharmacol. Ther..

[117] Heinig R., Fricke R., Wertz S., Nagelschmitz J., Loewen S. (2022). Results from Drug-Drug Interaction Studies In Vitro and In Vivo Investigating the Inhibitory Effect of Finerenone on the Drug Transporters BCRP, OATP1B1, and OATP1B3. Eur. J. Drug Metab. Pharmacokinet..

[118] Lehtisalo M., Tarkiainen E.K., Neuvonen M., Holmberg M., Kiiski J.I., Lapatto-Reiniluoto O., Filppula A.M., Kurkela M., Backman J.T., Niemi M. (2024). Ticagrelor Increases Exposure to the Breast Cancer Resistance Protein Substrate Rosuvastatin. Clin. Pharmacol. Ther..

[119] Choi H.G., Kwon B.-C., Kwon M.J., Kim J.H., Kim J.-H., Park B., Lee J.W. (2022). Association between Gout and Dyslipidemia: A Nested Case–Control Study Using a National Health Screening Cohort. J. Pers. Med..

[120] Lehtisalo M., Keskitalo J.E., Tornio A., Lapatto-Reiniluoto O., Deng F., Jaatinen T., Viinamaki J., Neuvonen M., Backman J.T., Niemi M. (2020). Febuxostat, But Not Allopurinol, Markedly Raises the Plasma Concentrations of the Breast Cancer Resistance Protein Substrate Rosuvastatin. Clin. Transl. Sci..

[121] Abed W., Abujbara M., Batieha A., Ajlouni K. (2022). Statin Induced Myopathy Among Patients Attending the National Center for Diabetes, endocrinology, & genetics. Ann. Med. Surg..

[122] Trivedi A., Sohn W., Kulkarni P., Jafarinasabian P., Zhang H., Spring M., Flach S., Abbasi S., Wahlstrom J., Lee E. (2021). Evaluation of drug-drug interaction potential between omecamtiv mecarbil and rosuvastatin, a BCRP substrate, with a clinical study in healthy subjects and using a physiologically-based pharmacokinetic model. Clin. Transl. Sci..

[123] Wang Z., Li Y., He X., Fu Y., Li Y., Zhou X., Dong Z. (2023). In vivo evaluation of the pharmacokinetic interactions between almonertinib and rivaroxaban, almonertinib and apixaban. Front. Pharmacol..

[124] Zhang Y., Shipkova P.A., Warrack B.M., Nelson D.M., Wang L., Huo R., Chen J., Panfen E., Chen X.Q., Fancher R.M. (2023). Metabolomic Profiling and Drug Interaction Characterization Reveal Riboflavin As a Breast Cancer Resistance Protein-Specific Endogenous Biomarker That Demonstrates Prediction of Transporter Activity In Vivo. Drug Metab. Dispos..

[125] Xie Y., Shao Y., Gong X., Wang M., Chen Y. (2023). Evaluation of P-glycoprotein-targeting circulating microRNAs as peripheral biomarkers for medically intractable epilepsy. Acta Epileptol..

[126] Roth M., Obaidat A., Hagenbuch B. (2012). OATPs, OATs and OCTs: The organic anion and cation transporters of the SLCO and SLC22A gene superfamilies. Br. J. Pharmacol..

[127] Li T.T., An J.X., Xu J.Y., Tuo B.G. (2019). Overview of organic anion transporters and organic anion transporter polypeptides and their roles in the liver. World J. Clin. Cases.

[128] Bruyere A., Le Vee M., Jouan E., Molez S., Nies A.T., Fardel O. (2021). Differential in vitro interactions of the Janus kinase inhibitor ruxolitinib with human SLC drug transporters. Xenobiotica.

[129] Hwang S., Lee Y., Jang Y., Cho J.Y., Yoon S., Chung J.Y. (2024). Comprehensive Evaluation of OATP- and BCRP-Mediated Drug-Drug Interactions of Methotrexate Using Physiologically-Based Pharmacokinetic Modeling. Clin. Pharmacol. Ther..

[130] Yamazaki T., Desai A., Goldwater R., Han D., Lasseter K.C., Howieson C., Akhtar S., Kowalski D., Lademacher C., Rammelsberg D. (2017). Pharmacokinetic Interactions Between Isavuconazole and the Drug Transporter Substrates Atorvastatin, Digoxin, Metformin, and Methotrexate in Healthy Subjects. Clin. Pharmacol. Drug Dev..

[131] Motoki K., Taniguchi T., Ashizawa N., Sakai M., Chikamatsu N., Yamano K., Iwanaga T. (2023). Uricosuric Agents Affect Plasma and Kidney Concentration of Adefovir via Inhibition of Oat1 and Mrp2 in Rats. Biol. Pharm. Bull..

[132] Wu T., Li H., Chen J., Cao Y., Fu W., Zhou P., Pang J. (2017). Apigenin, a novel candidate involving herb-drug interaction (HDI), interacts with organic anion transporter 1 (OAT1). Pharmacol. Rep..

[133] Clarke R., Leonessa F., Trock B. (2005). Multidrug resistance/P-glycoprotein and breast cancer: Review and meta-analysis. Semin. Oncol..

[134] Zhu S., Sun C., Cai Z., Li Y., Liu W., Luan Y., Wang C. (2024). Effective therapy of advanced breast cancer through synergistic anticancer by paclitaxel and P-glycoprotein inhibitor. Mater. Today Biol..

[135] Zhou X., Zhang P., Yang Y., Shi W., Liu L., Lai Z., Zhang X., Pan P., Li L., Du J. (2024). Highly Potent and Intestine Specific P-Glycoprotein Inhibitor to Enable Oral Delivery of Taxol. Angew. Chem. Int. Ed..

[136] Loos N.H.C., Martins M.L.F., de Jong D., Lebre M.C., Tibben M., Beijnen J.H., Schinkel A.H. (2024). Coadministration of ABCB1/P-glycoprotein inhibitor elacridar improves tissue distribution of ritonavir-boosted oral cabazitaxel in mice. Int. J. Pharm..

[137] Zhang Y., Li C., Xia C., Wah To K.K., Guo Z., Ren C., Wen L., Wang F., Fu L., Liao N. (2022). Adagrasib, a KRAS G12C inhibitor, reverses the multidrug resistance mediated by ABCB1 in vitro and in vivo. Cell Commun. Signal..

[138] Yang Q., To K.K.W., Hu G., Fu K., Yang C., Zhu S., Pan C., Wang F., Luo K., Fu L. (2024). BI-2865, a pan-KRAS inhibitor, reverses the P-glycoprotein induced multidrug resistance in vitro and in vivo. Cell Commun. Signal..

[139] Marcelletti J.F., Sikic B.I. (2024). Continuous 72-h infusion of zosuquidar with chemotherapy in patients with newly diagnosed acute myeloid leukemia stratified for leukemic blast P-glycoprotein phenotype. Cancer Chemother. Pharmacol..

[140] Sun D., Liu J., Wang Y., Dong J. (2022). Co-administration of MDR1 and BCRP or EGFR/PI3K inhibitors overcomes lenvatinib resistance in hepatocellular carcinoma. Front. Oncol..

[141] Deng F., Sjostedt N., Santo M., Neuvonen M., Niemi M., Kidron H. (2023). Novel inhibitors of breast cancer resistance protein (BCRP, ABCG2) among marketed drugs. Eur. J. Pharm. Sci..

[142] Jackson S.M., Manolaridis I., Kowal J., Zechner M., Taylor N.M.I., Bause M., Bauer S., Bartholomaeus R., Bernhardt G., Koenig B. (2018). Structural basis of small-molecule inhibition of human multidrug transporter ABCG2. Nat. Struct. Mol. Biol..

[143] Zechner M., Castro Jaramillo C.A., Zubler N.S., Taddio M.F., Mu L., Altmann K.H., Kramer S.D. (2023). In Vitro and In Vivo Evaluation of ABCG2 (BCRP) Inhibitors Derived from Ko143. J. Med. Chem..

[144] Vesga L.C., Kronenberger T., Tonduru A.K., Kita D.H., Zattoni I.F., Bernal C.C., Bohorquez A.R.R., Mendez-Sanchez S.C., Ambudkar S.V., Valdameri G. (2021). Tetrahydroquinoline/4,5-Dihydroisoxazole Molecular Hybrids as Inhibitors of Breast Cancer Resistance Protein (BCRP/ABCG2). ChemMedChem.

[145] Antoni F., Bause M., Scholler M., Bauer S., Stark S.A., Jackson S.M., Manolaridis I., Locher K.P., Konig B., Buschauer A. (2020). Tariquidar-related triazoles as potent, selective and stable inhibitors of ABCG2 (BCRP). Eur. J. Med. Chem..

[146] Kanai Y., Clemencon B., Simonin A., Leuenberger M., Lochner M., Weisstanner M., Hediger M.A. (2013). The SLC1 high-affinity glutamate and neutral amino acid transporter family. Mol. Asp. Med..

[147] Johnston R.A., Rawling T., Chan T., Zhou F., Murray M. (2014). Selective inhibition of human solute carrier transporters by multikinase inhibitors. Drug Metab. Dispos..

[148] Bruyere A., Hubert C., Le Vee M., Chedik L., Sayyed K., Stieger B., Denizot C., Parmentier Y., Fardel O. (2017). Inhibition of SLC drug transporter activities by environmental bisphenols. Toxicol. In Vitro.

[149] Puris E., Fricker G., Gynther M. (2023). The Role of Solute Carrier Transporters in Efficient Anticancer Drug Delivery and Therapy. Pharmaceutics.

[150] Boeszoermenyi A., Bernaleau L., Chen X., Kartnig F., Xie M., Zhang H., Zhang S., Delacretaz M., Koren A., Hopp A.K. (2023). A conformation-locking inhibitor of SLC15A4 with TASL proteostatic anti-inflammatory activity. Nat. Commun..

[151] Chen X., Xie M., Zhang S., Monguio-Tortajada M., Yin J., Liu C., Zhang Y., Delacretaz M., Song M., Wang Y. (2023). Structural basis for recruitment of TASL by SLC15A4 in human endolysosomal TLR signaling. Nat. Commun..

[152] Rives M.L., Javitch J.A., Wickenden A.D. (2017). Potentiating SLC transporter activity: Emerging drug discovery opportunities. Biochem. Pharmacol..

[153] Shchulkin A.V., Abalenikhina Y.V., Slepnev A.A., Rokunov E.D., Yakusheva E.N. (2023). The Role of Adopted Orphan Nuclear Receptors in the Regulation of an Organic Anion Transporting Polypeptide 1B1 (OATP1B1) under the Action of Sex Hormones. Curr. Issues Mol. Biol..

[154] Buxhofer-Ausch V., Secky L., Wlcek K., Svoboda M., Kounnis V., Briasoulis E., Tzakos A.G., Jaeger W., Thalhammer T. (2013). Tumor-specific expression of organic anion-transporting polypeptides: Transporters as novel targets for cancer therapy. J. Drug Deliv..

[155] Verscheijden L.F.M., Koenderink J.B., de Wildt S.N., Russel F.G.M. (2021). Differences in P-glycoprotein activity in human and rodent blood-brain barrier assessed by mechanistic modelling. Arch. Toxicol..

